# Health problems of Nepalese migrants working in three Gulf countries

**DOI:** 10.1186/1472-698X-11-3

**Published:** 2011-03-28

**Authors:** Suresh Joshi, Padam Simkhada, Gordon J Prescott

**Affiliations:** 1 School of Medicine and Dentistry, Section of Population Health, University of Aberdeen, Polwarth Building, Foresterhill, Aberdeen, UK; 2 School of Health and Related Research (ScHARR), University of Sheffield, Regent Court, Sheffield, UK

## Abstract

**Background:**

Nepal is one of the largest suppliers of labour to countries where there is a demand for cheap and low skilled workers. In the recent years the Gulf countries have collectively become the main destinations for international migration. This paper aims to explore the health problems and accidents experienced by a sample of Nepalese migrant in three Gulf countries.

**Methods:**

A cross-sectional survey was conducted among 408 Nepalese migrants who had at least one period of work experience of at least six months in any of three Gulf countries: Qatar, Saudi Arabia and United Arab Emirates (UAE). Face to face questionnaire interviews were conducted applying a convenience technique to select the study participants.

**Results:**

Nepalese migrants in these Gulf countries were generally young men between 26-35 years of age. Unskilled construction jobs including labourer, scaffolder, plumber and carpenter were the most common jobs. Health problems were widespread and one quarter of study participants reported experiencing injuries or accidents at work within the last 12 months. The rates of health problems and accidents reported were very similar in the three countries. Only one third of the respondents were provided with insurance for health services by their employer. Lack of leave for illness, cost and fear of losing their job were the barriers to accessing health care services. The study found that construction and agricultural workers were more likely to experience accidents at their workplace and health problems than other workers.

**Conclusion:**

The findings suggest important messages for the migration policy makers in Nepal. There is a lack of adequate information for the migrants making them aware of their health risks and rights in relation to health services in the destination countries and we suggest that the government of Nepal should be responsible for providing this information. Employers should provide orientation on possible health risks and appropriate training for preventive measures and all necessary access to health care services to all their workers.

## Background

Migration of people has been a common phenomenon since the beginning of human civilization. With increasing numbers of people moving from one country to another, migrants' health has become a key global public health issue [1]. There is a high chance that low-skilled migrants from low income countries work in risk prone working conditions since they usually accept the jobs that are rejected by local workers [2,3]. Industrialized countries are interested in the recruitment of migrants from poor countries for physical labour and the migrants are often provided with short term contracts [4,5]. Marginalized groups such as migrants or ethnic minority groups often have inadequate access to health care services with poor provision of health services, inconvenient location of health services and cultural differences being the barriers to access to health care [6-8]. South Asian countries are the main suppliers of migrant workers to the Gulf countries [2,9-11].

### Migration context and trends in Nepal

The migration of Nepalese people for foreign employment began early in the nineteenth century. The perception of Nepalese migrants has radically shifted from "Global warriors to Global workers" [12] during the last few decades. Existing poverty, limited employment opportunities, deteriorating agricultural productivity and armed conflict are some of the reasons behind international labour migration [13]. Most rural households in Nepal depend on the earnings of at least one family member who is employed away from home [14,15]. Nepalese migrants, especially from middle or low class families, are migrating temporarily to different countries [16,17]. It has been estimated that in recent years more than 500 Nepalese people per day go abroad for foreign employment [12-14,18].

Nepal Institute of Development Studies (NIDS) found that two-thirds of Nepali working overseas were employed in the Gulf countries, mainly in Saudi Arabia (42%), Qatar (11.5%) and United Arab Emirates (9%) [15]. Most of the Nepalese migrants in the Gulf countries are involved in heavy manual labour on road building sites or construction sites, often in high temperatures [18]. The Government of Nepal prohibited new registrations of female migrants for travelling to work in the Gulf countries in 1998 to ensure against the physical and sexual abuse. As the Government of Nepal has lifted the ban in 2003, female migrants are allowed to work in the organised sector such as in hospitals, hotels and shops [13].

### Health problems and accidents

Most international migrants particularly in Gulf countries are employed in the occupations which fall in the category of "three Ds" (difficult, dirty, and dangerous) [15,18-20]. The contractors in the construction sector often do not meet the labour laws and health and safety standards in order to reduce the maintenance cost of the workforce [21].

The Government of Nepal has introduced mandatory pre-departure training for the migrant workers, but some of the training institutes in Nepal provide certificates to the migrants without completing the training or even after no attendance at the orientation session at all [15]. Nepalese migrants have an increased chance of accidents and suffering from other health problems than the local workers because of the long working hours, local languages, and poor living and working conditions. There have also been some reported cases of unexplained deaths of Nepalese migrants [15,22]. Health and psychological problems due to degrading and dangerous jobs have forced some Nepalese migrants to leave their jobs and return home [13,22].

Migrants are more likely to suffer from occupational injuries and disability than the native workers [6,23,24]. Migrants involved in construction, mining, agricultural work and manufacturing are often exposed to a range of occupational risks including exposure to toxic agents and lack protective clothing or other equipment [25,26].

Temporary migrants are usually excluded from the health, welfare and social services at the destination country [1,6,27-29]. Migrants usually have low capacity to pay for medical services, poor access to health care and unsatisfactory health outcomes [24,30]. Costs for health care services are often expensive for migrants and sometimes migrants even have to deposit certain amounts of money to receive health services [31]. Nepalese migrants to the Gulf countries and their family members typically do not have insurance coverage [22]. Legal migrants are only insured for the accidents at the work place and they find it hard to access the health services for other health problems [15,19,32].

This study aimed to explore the health problems experienced by a sample of Nepalese migrants working in three Gulf countries; Qatar, Saudi Arabia and United Arab Emirates (UAE). The study approach was specifically focused on investigating the number and type of health problems and accidents experienced by these migrant during their stay abroad. Additionally, the study aimed to find out about the health care services used by migrant workers within those Gulf countries and also explore the barriers to health care services. The findings of this survey could be beneficial to design effective information, education and communication (IEC) interventions for the Nepalese migrants working in the Gulf countries as well as in other similar regions.

## Methods

This study was a cross-sectional survey of the self-reported health of Nepalese migrants during their stay abroad. A face to face structured questionnaire interview was used to obtain the data. A formal probability sampling procedure was difficult to use for this study due to the lack of a sampling frame including the total number of migrants who were working in the Gulf countries. It was possible to obtain the estimated recorded lists of migrants from the government organisations and other manpower agencies, but it was considered too difficult to recruit the respondents from these lists because they are such a highly mobile population. Therefore convenience sampling was used to recruit the participants for this survey.

The data were collected in March and April 2009 from two different types of location: the International Airport and nearby hotels/lodges. Travelling by air is the only route by which Nepalese migrants can to go to the Gulf countries. There is only one international airport in Nepal, Tribhuvan International Airport of Nepal, located in Kathmandu valley. Different national as well as international airlines have regular scheduled flights to the different Gulf countries. Participants recruited at the airport were interviewed at the departure area, where normally Nepalese migrant travellers come three to four hours before their scheduled flight time.

Additionally, hotels and lodges near the airport and the bus park area of Kathmandu were also approached for permission to interview the travellers. The processing of visas and medical check ups are only possible in Kathmandu, so the people planning to migrate to the Gulf countries have to stay for several days in Kathmandu valley. By recruiting the study population from both of these types of site we were able to interview migrants who had just returned from Gulf countries and migrants who were about to go to those countries after a stay in Nepal. It was assumed that it would be possible to meet the migrants from many different socio-economic backgrounds from the different regions of Nepal at the same place.

The inclusion criteria for the study were: adult people who had work experience of at least six months in one of the three Gulf countries (Qatar, Saudi Arabia and United Arab Emirates (UAE)), who were in Nepal at the time of recruitment and who had returned to Nepal within last 12 months (on their annual leave or for any other reason).

We were not able to identify a previously validated, standardised questionnaire suitable for recording self reported health problems of Nepalese migrants in similar settings. We developed the questionnaire on the basis of previous qualitative literature and the opinions of the subject experts in this area. Some of the demographic questions were adopted from a previous study of Nepalese migrants in the UK [33]. Key informants for this research were immigration officers, manpower agencies personnel and hotel owners during the initial phase of questionnaire design. The questionnaire was piloted among the respondents who had previous work experience for more than a six months period in the Gulf countries. Our main study data did not include the data obtained from the respondents of the pilot study or the information obtained from the key informants.

Ethical approval was obtained from the Ethics committee of Nepal Health Research Council (NHRC). Before starting the research project, approval and permission were gained from the Immigration Department, Tribhuvan International Airport, Kathmandu and from the owners of the hotels near the airport and bus park area. Verbal consent was obtained from the potential participants after they had received this information. The interview started only if consent was given.

There was no published information which could be used to estimate the percentage of Nepalese migrants with access to health care services in the Gulf countries. However, if we assumed that 50% of Nepalese migrants might have access to health care, for a power calculation this percentage would require the largest sample for any given width of 95% confidence interval. With a sample of 400 a population percentage of 50% could be estimated with a 95% confidence interval of 45% to 55%.

Effort was made by the interviewers to ensure that the questionnaires were complete as possible. The raw data were checked and, where possible, errors were corrected. Statistical analysis was carried out by using SPSS version 17. Open ended answers were recorded as free text and were recoded later after identifying the main themes and their frequencies.

The principle outcome variables in the analysis were the migrants' self-reported health problems and the accidents of the migrants during their last 12 months of stay abroad. The self-reported questions for the outcome variables were: Did you suffer from any health problem during your last 12 months of stay in that region? Did you have any type of injury or accident at your workplace during last 12 months of stay abroad? The last primary job of the respondents were recoded into categories: construction work, driving, wholesale and retail trade, agricultural and farm work, household work, hotel and restaurant work, and clerical work. The type of accidents at work were also recoded as burns, falls, cuts, motor vehicle accidents, fractures and dislocations, and faints/temperature related accidents. Self-reported health problems suffered during the most recent working time in the Gulf countries (within the last 12 months) were also recoded on the basis of common medical conditions. Variables such as educational status, ethnicity, marital status and types of health problems were grouped into fewer categories than were printed on the questionnaire to avoid very small counts of respondents in some categories.

Descriptive statistics were used to explore the demographic variables and the health problems. Counts and percentages were used to describe categorical data. Chi-square tests were applied to compare proportions with health problems and accidents between job type and destination country. Multiple logistic regression analysis was used to investigate the multiple factors related to the principal outcomes. Potential confounding variables such as age, educational status, marital status, destination country, last primary occupation last residence, training for health risk were considered in the multivariable analyses.

## Results

### Demographic characteristics of the respondents

The total number of participants surveyed was 408. Out of the respondents only 31 (7.6%) were female. The mean and standard deviation (SD) of age of the respondents were 32 and 6.5 years respectively. The age range was 18 to 53 years old with 53.4% of respondents between 26-35 years old. More than half of the respondents 209 (51.2%) were from hill regions of Nepal. The vast majority 359 (88%) of the respondents were Hindu and 163 (40%) were of Brahmin/Chhetri ethnic background.

All of the respondents in this study were able to read and write. The majority of the respondents 311 (76.2%) had only completed their primary education including through the various informal education systems in Nepal. Only 28 (6.9%) of the respondents had completed their higher education level beyond SLC (School Leaving Certificate). The majority of the respondents were married (80.6%). Only 3 of the respondents (0.7%) were living with their spouse or partner during their stay abroad (Table 1).

**Table 1:** Demographic characteristic of the respondents

Variables	Frequency (n = 408)	Percentage (%)
**Gender**		
Male	377	92.4
Female	31	7.6

**Age group (years)**		
18-25	77	18.9
26-35	218	53.4
> 35	113	27.7

**Caste/ethnicity**		
Brahmin/Chhetri	163	40.0
Gurung/Tamang	72	17.6
Magar/Rai/Limbu	56	13.7
Madhesis/Tharu	54	13.2
Others	63	15.4

**Birth district by ecological zone**		
Terai (Flat lands)	180	44.1
Hills	209	51.2
Mountains	19	4.7

**Education (n = 406)**		
Primary education	311	76.2
Secondary education (SLC)	69	16.9
Higher education/beyond SLC	28	6.9

**Marital status**		
Married/civil partner	329	80.6
Unmarried	76	18.6
Divorced/separated	3	0.7

**Country of work experience**		
Qatar	205	50.2
Saudi Arabia	137	33.6
United Arab Emirates (UAE)	66	16.2

**Last primary occupation during stay abroad**		
Construction work	224	54.9
Driving	24	5.9
Wholesale and retail trade	46	11.3
Agricultural and farm work	17	4.2
Household/manual servant	74	18.1
Clerical and related work	23	5.6

From Table 1, it can also be seen that half 205 (50.2%) of the total respondents were currently working or had at least six month experience in Qatar. The percentages of people working in Saudi Arabia and UAE were found to be 137 (33.6%) and 66 (16.2%). More than half of respondents, 224 (54.9%), were involved in various types of construction work such as: labourer, scaffolder, general helpers and others. The second most frequent occupation was found to household and manual activities, 74 (18.1%). Wholesale and retail, driving and clerical related occupations were also common among Nepalese migrant.

### Health problems and accidents and access to health care services

This survey found that more than half, 231 (56.6%), of the respondents suffered from some type of health problem during their last 12 months of stay abroad. Some people suffered from more than one type of health problem. Among the 231 respondents who experienced a health problem the most common types, in order, were headache or fever, 71 (30.7%), respiratory symptoms, 49 (21.2%), musculoskeletal problems, 46 (19.9%), gastrointestinal illness, 45 (19.5%), and injuries/poisoning, 32 (13.9%).

A quarter of the total respondents, 102 (25.0%), reported experiencing some type of injury or accident at their workplace during their last job. The survey found that some people experienced more than one type of accident. Different types of cuts, 41 (40.2%), and fractures or dislocations, 21 (20.6%), were the most common type of injuries and accidents that occurred during work among the respondents surveyed. Temperature related illness (17.6%) (such as heat stroke or faint) and other accidents and falls (11.8%) were also common.

The survey found that only one third of the respondents, 149 (36.5%), were insured for health services in the countries where they were working. All of those migrants who had health insurance coverage reported that the company/employer provided this insurance.

Out of the total respondents who suffered some type of health problem (231), the majority of these, 192 (83.1%), sought health services or treatment in the country where they were working. Some workers visited more than one place to receive health services. Of the respondents who visited at least one place for treatment, 115 (59.9%) had gone to a government hospital and 87 (45.3%) had gone to a private health centre. On site treatment, self-medication, pharmacy treatment and treatment from friends and relatives were less common. 39 (16.9% of those experiencing a health problem) did not seek health services. 29 of these (74.4% of 39) did not feel the need to seek treatment during illness. Almost half of those not seeking treatment, 19 (48.7%), reported that lack of provision of leave during the health problems was a barrier for accessing health services. Service fees and other expenses were also found to be barriers.

We did not find the statistically significant differences between the three working countries and the likelihood of health problems (p = 0.7) or accidents (p = 0.6) during the stay abroad (Table 2).

**Table 2:** Association between working countries and health problems and accidents

Working countries	Health problems suffered during last 12 month		Total
		
	Yes (%)	No (%)	
Qatar	120 (58.5)	85 (41.5)	205

Saudi Arabia	74 (54.0)	63 (46.0)	137

United Arab Emirates	37 (56.1)	29 (43.9)	66

Total	231 (56.6)	177 (43.4)	408

**Working countries**	**Accident at the work place**		**Total**
		
	**Yes (%)**	**No (%)**	

Qatar	47 (22.9)	158 (77.1)	205

Saudi Arabia	36 (26.3)	101 (73.7)	137

United Arab Emirates	19 (28.8)	47 (71.2)	66

Total	102 (25.0)	306 (75.0)	408

There were significant differences in the percentages experiencing health problems during the last 12 months between the different last primary jobs of the respondents (Chi-square, p = 0.03) (Table 3). Most of the agricultural or farm workers 14/17 (82.4%) reported that they suffered from health problems during last 12 months of their stay abroad compared with smaller percentages of household/ manual servants 32/74 (43.2%) and clerical workers 10/23 (43.5%). The percentage of people suffering health problems was also similarly high at close to 60% among construction workers, drivers and those in the retail trades.

**Table 3:** Association between last primary job and health problems and accidents

Last primary job	Health problems suffered during last 12 months		Total
		
	Yes (%)	No (%)	
Construction work	134 (59.8)	90 (40.2)	224

Driving	14 (58.3)	10 (41.7)	24

Wholesale and retail trade	27 (58.7)	19 (41.3)	46

Agriculture and farm work	14 (82.4)	3 (17.6)	17

Household/ manual servant	32 (43.2)	42 (56.8)	74

Clerical and related work	10 (43.5)	13 (56.5)	23

Total	231 (56.6)	177 (43.4)	408

**Last primary job**	**Accident at the work place**		**Total**
		
	**Yes (%)**	**No (%)**	

Construction work	75 (33.5)	149 (66.5)	224

Driving	5 (20.8)	19 (79.2)	24

Wholesale and retail trade	3 (6.5)	43 (93.5)	46

Agriculture and farm work	7 (41.2)	10 (58.8)	17

Household/ manual servant	10 (13.5)	64 (86.5)	74

Clerical and related work	2 (8.7)	21 (91.3)	23

Total	102 (25.0)	306 (75.0)	408

From Table 3, it can also be seen that construction workers, 75/224 (33.5%), and agricultural or farm workers, 7/17 (41.2%), were more likely to have had an accident at work compared to wholesale and trade workers, 3/43 (6.5%), and clerical related workers, 2/23 (8.7%). The last primary job and the likelihood of accidents at the work place were found to be significantly associated (p < 0.001).

### Determinants of health problems and accidents

Multivariate binary logistic regression models were constructed for the outcome of health problems with predictor variables such as age, birth regions, educational status, marital status, working country and primary occupation. None of these variables were significantly associated with the occurrence of health problems after adjustment for all the others. Lower proportions of workers of older ages had suffered from health problems compared to workers of 18-25 years old, but the odds of suffering from health problems were not significantly different among the three age groups. Similarly, people who had completed their secondary or higher education had lower odds of suffering from health problems than people who had only completed only primary education (OR = 0.63, 95%CI = 0.38 to 1.29), but not significantly so. The odds of suffering from health problems were similar in the three Gulf countries. Agricultural workers had higher odds of suffering health problems than the construction workers (OR = 3.21, 95%CI = 0.88 to 11.76), but the small numbers of agricultural workers led to very wide confidence intervals (Table 4).

**Table 4:** Multivariate logistic regression model for the determinants of health problems

			95% C.I.	
			
Variables	P-value	Odds ratio	Lower	Upper
**Age group**	0.75^(b)^			
18-25^(a)^		1.00		
26-35		0.94	0.49	1.81
> 35		0.78	0.37	1.68

**Birth region**	0.06^(b)^			
Terai^(a)^		1.00		
Hills		0.80	0.53	1.22
Mountains		3.60	0.99	13.05

**Education status**	0.09^(b)^			
Primary education(a)		1.00		
Secondary education (SLC) and beyond SLC		0.63	0.38	1.06

**Marital status**	0.23^(b)^			
Married^(a)^		1.00		
Others		0.66	0.34	1.29

**Working county**	0.35(b)			
Qatar^(a)^		1.00		
Saudi Arabia		0.70	0.43	1.14
United Arab Emirates (UAE)		0.94	0.52	1.69

**Primary occupation**	0.12^(b)^			
Construction work^(a)^		1.00		
Driving		1.07	0.44	2.60
Wholesale and retail trade		1.10	0.55	0.22
Agricultural and farm work		3.21	0.88	11.76
Household/manual servant		0.58	0.32	1.02
Clerical and related work		0.57	2.22	1.57

a: this indicates the reference group

b: this indicates the p-value for whole variable in the model

The multivariate analysis (Table 5), showed that people of 26-35 or >35 years old had higher odds of experiencing an accident compared to the people of 18-25 years of age. Additionally workers who had completed secondary level or higher level of education had lower odds of experiencing an accident than people who had only completed primary level of education. The odds of accidents among the people who had work experience in UAE was 1.8 times greater than people who had work experience in Qatar, but this elevation of the odds was not statistically significant.

**Table 5:** Multivariate logistic regression model for the determinants of accidents

			95% C.I.	
			
Variables	P-value	Odds ratio	Lower	Upper
**Age group**	0.40^(b)^			
18-25^(a)^		1.00		
26-35		1.58	0.80	3.14
> 35		1.32	0.62	2.80

**Education status**	0.14^(b)^			
Primary education^(a)^		1.00		
Secondary education (SLC) and beyond SLC		0.60	0.31	1.19

**Working county**	0.21^(b)^			
Qatar^(a)^		1.00		
Saudi Arabia		1.17	0.68	2.02
United Arab Emirates (UAE)		1.85	0.94	3.63

**Primary occupation**	< 0.001^(b)^			
Construction work^(a)^		1.00		
Driving		0.44	0.15	1.26
Wholesale and retail trade		0.14	0.04	0.47
Agricultural and farm work		1.37	0.49	3.81
Household/manual servant		0.31	0.15	0.66
Clerical and related work		0.23	0.05	1.06

**Training before job**	0.29^(b)^			
Yes^(a)^		1.00		
No		1.30	0.80	2.13

a: this indicates the reference group

b: this indicates the p-value for whole variable in the model

Relative to construction workers, agricultural workers had higher odds of experiencing accidents whereas workers in other occupations had significantly lower odds of experiencing accidents. Low numbers of workers in some occupations may limit the generalisability of this finding. Lack of training before the job was associated with a non-significant increase in the odds of accidents (OR = 1.30, 95%CI = 0.80 to 2.13) (Table 5).

## Discussion

The annual report of Department of Labour (DoL), Nepal found 169,932 migrants registered to work in the three Gulf countries during the fiscal year of 2007/2008. The percentage of male and female was found to be 169,840 (99.9%) and 92 (0.1%) respectively. Out of these the distributions of Nepalese migrants to the three Gulf countries were: Qatar (49.3%), UAE (25.7%) and Saudi Arabia (24.8%) [34]. In comparison to our study the distribution of migrants across these countries was fairly similar. In the case of Saudi Arabia (33.2%) our study found a higher percentage of people working in that country compared to the percentage of data provided by the Government. The slight difference in the proportions in three different countries in our study and the Department of Labour may be because our inclusion and exclusion criteria meant that we have interviewed people whose experience extended back more than 6 months to several years, rather than just the most recent 12 months. We might also have interviewed some people working illegally in those countries who would not be represented in Government figures. We were able to interview only 31 females for this study. It was difficult to find female respondents in our study area because Government of Nepal has banned the sending of newly registered Nepalese female labour workers to the Gulf countries. Typically the female migrants interviewed had returned to Nepal from a Gulf country and were planning to return to the Gulf via India. The small number of females interviewed may not be representative of all the Nepalese female migrants working in the Gulf countries. Their illegal migration status may have had influenced the willingness of women to state their true destination country and to participate in this study. The true percentage of female migrants travelling to these three Gulf countries may be very different to that reported in this study.

Nepalese migrants are involved in various types of occupations in Gulf countries including those in catering or domestic work, such as waiter or juice maker. The majority of Nepalese migrants are low skilled labourers with only a few skilled workers who are engineers [12]. The respondents of our study were involved in very similar types of jobs. The highest educational qualification of the majority of the Nepalese migrants in our study was at primary level. The studies by Graner and Gurung also reported SLC failure or dropout for most of the Nepalese migrants in Gulf countries [12,22]. Our study found a significant association between education status and the last primary occupation (p < 0.001) with people who had lower education being more likely to have worked in unskilled jobs in construction, or farm work during their stay abroad.

We frequently hear tragic news about accidents, disabilities, violence towards and deaths of Nepalese migrants in the Gulf countries from national as well as international newspapers. The government of Nepal appears to be primarily concerned with ensuring a steady income from remittances by sending people to those regions, whereas companies of Gulf countries are in need of cheap manpower ignoring the workers' health and well being [15].

More than half of the migrants in this study had experienced health problems. There was a significant association between the primary occupation and the likelihood of health problems of the respondents during their stay abroad, but this did not remain statistically significant after adjustment for other potentially confounding variables.

In the previous study by NIDS (2006), the reported health problems of the Nepalese migrants in the Gulf countries and Malaysia were stomach pain, fever, malaria, jaundice, blood pressure, obesity, physical disability, temperature related illness, kidney failure and mental trauma. Some of the respondents in our study had also reported the diseases mentioned above whereas the health problems listed in the NIDS study were obtained from the qualitative in-depth interview without grouping into disease type. Similarly, a study conducted in UAE which had 90% of respondents from the Indian subcontinent found 44.0% of the farm workers suffered from headache and 36.9% suffered from respiratory symptoms [35]. In contrast in our study, 28.6% of the 14 agriculture or farm workers suffered from fever and headache and 21.4% suffered from respiratory symptoms. The much higher percentages for both of the health problems reported in the UAE study may have been due to higher exposures to agricultural chemicals experienced at that time, but since we have no objective measure of exposures or illness we cannot be certain. The health problems of our study were self-reported whereas in the previous study chronic respiratory symptoms were recorded by using the database of British Research Council and other validated questionnaires.

In the fiscal year 2008/2009, the nationwide major causes of out-patient department (OPD) morbidity in Nepal were reported as intestinal worms, acute respiratory infection, pyrexia of unknown origin, gastritis, headache/migrane and falls/injuries/fractures respectively [36]. The national figures of health problems include some of the diseases suffered by Nepalese migrant of our study. But it is inappropriate to compare our findings with the national data because the cases of morbidity reported in Nepal were diagnosed by health care provider whereas our study includes only the self-reported health problems. Additionally, the health problems reported in Nepal were not classified in terms of age, sex and occupation but in our study the majority were males of 26-35 years and most of them involved in unskilled construction jobs.

A quarter of migrants in our study experienced accidents at their work place during their last 12 months of stay abroad. Cuts and fractures were the most common type of injuries at the workplace. Nepalese migrants labourers are insufficiently protected from industrial accidents and are frequently sent back to Nepal once injured [13,22]. A study in Bahrain including a majority of non-Bahraini workers found 56.6% to 54.1% of the construction workers experienced an occupational injury according to records from 1988-1991. The major causes of accidents were being struck by flying objects, penetration by a sharp object, cuts and falls [23]. In contrast, our study found a lower percentage, 33.5%, of construction workers reported having experienced injuries or accidents at their workplace. The major causes of accidents were fairly similar despite the classification of injuries being different in our study. The previous study analysed all the records of occupational injuries for the period 1988-1991, whereas the percentage in our study is based on the self reporting of work place accidents.

Due to our method of data collection and inclusion criteria, there is a selection bias since we cannot have any reports of accidents or illnesses which led to death, or serious accidents or illnesses such that the worker could not complete 6 months of work abroad. It is also very likely that any worker who had experienced any injury or illness serious enough to limit their ability to migrate for work again would have been missed by this study. There is also the potential for recall bias. By asking about self reported health problems during their last 12 months of stay abroad this limited the length of time that respondents had to remember. However, we recognise that some individuals who had returned from the Gulf only just under 12 months ago would meet the inclusion criteria, but would be attempting to remember health events from almost 24 months ago to almost 12 months ago.

Nepalese migrants are often covered with insurance only for first year of work abroad. The company only covers the cost for accidents and deaths at the workplace [22]. This previous study supports the findings of our study in which only one third of the respondents were insured for health services. For all migrants, their pre-visa documents for work permits to go to the Gulf countries are processed by the recruitment agencies in Nepal [15,18,22], so the workers might not be aware about matters relating to insurance. The majority of respondents in our study have only completed primary education and are unskilled or semi-skilled labourers, which supports the previous evidence that states they are commonly unaware about their own status in relation to health insurance. Other reasons for the low coverage of health insurance in this study may be the cost and the illegal status of some Nepalese migrant workers.

The Government hospitals are the most common places for migrants to seek health services in the Gulf countries and that was supported by the findings of our study. Migrants must have a work permit to seek treatment from Government hospitals [19]. In this study we were not able to identify the legal status of the respondents, so we are not able to confirm whether the legal status was a barrier to accessing health services. In our study, out of the respondents who did not seek treatment during illness, the majority perceived no need for treatment. So the perception about the severity of the health problem might have influenced the health seeking behaviour of the respondents.

There was possibly a healthy worker effect in the selection of participants. Nepalese migrants who had worked in these countries for more than five years in total were slightly more likely to have suffered from health problems than those who had stayed there for a shorter time. The likelihood of experiencing some injuries at work was slightly higher for the migrants who had stayed up to two years compared to those who had stayed longer. However, neither association was statistically significant.

The statistically significant association between the last primary occupation and the odds of accidents at the work place shows that Nepalese migrants working in the construction and agricultural sectors were at higher risk than other occupational groups. Results from the multiple logistic regression analyses did not identify any group as particularly likely to experience health problems and after adjusting for possible confounding factors only primary occupation was associated with accidents. The questionnaire only recorded information on primary occupation, so if the respondent changed occupation during the last 12 month period of work abroad, the analyses may relate some health events to the wrong occupations.

## Conclusions

Health problems were widespread and one quarter of Nepalese migrant workers had experienced injuries or accidents at work within the last 12 months of stay abroad. Headache, respiratory diseases, gastrointestinal diseases, musculoskeletal diseases and injuries were the most common health problems experienced by them. Additionally, different types of cuts and fracture or dislocation were the most common type of injuries and accidents that occurred during work among the migrants. Although the majority of the Nepalese migrants were employed in risky jobs, most of them were not provided training for prevention or management of health risks before their work or provided preventive measures during work. From this study we can say that no country was especially risky and no group of workers were identified as especially vulnerable to health problems or accidents. A simple recommendation could be made to the policymakers of the Government of Nepal to take responsibility for Nepalese migrants in terms of making them aware of their health risks and rights in relation to health services in the destination countries. Involvement of migrant workers and their representatives in meetings with government and employers to discuss issues relating to migrant workers and new policies might empower the migrant workers. There is need to promote the right to freely associate and to organize and bargain collectively throughout the Gulf countries. Independent trade unions can take care of many of the issues of migrant workers by advocating for their rights and engaging employers and governments through the accepted methods. Similarly, campaigns to increase awareness of legal reforms and workers rights throughout the Gulf countries would help to improve the migrant's working conditions.

Employers should be responsible for ensuring that some form of health services are available, accessible and affordable to all migrant workers during their illnesses and other health problems. Insurance for health services should be provided to all migrants for the duration of their stay abroad rather than just one year. Another possible solution to protect the migrant might be the provision of training for preventive health measures for health risks before they start the jobs and also the employers providing protective clothing and equipment to all the workers.

## Competing interests

The authors declare that they have no competing interests.

## Authors' contributions

All authors are equally involved in the conception and design of study. SJ conducted the data collection, interpretation and drafted the manuscript. PPS and GJP provided comments and approved the final manuscript. All authors read and approved the final version of the manuscript.

## Pre-publication history

The pre-publication history for this paper can be accessed here:

http://www.biomedcentral.com/1472-698X/11/3/prepub
